# Asthma and Obstructive Sleep Apnea Overlap Syndrome Identifies a Phenotype of Sleep Instability and Increased Psychological Burden

**DOI:** 10.3390/life16040686

**Published:** 2026-04-18

**Authors:** Antonio Fabozzi, Izolde Bouloukaki, Violeta Moniaki, Eleni Mavroudi, Matteo Bonini, Paolo Palange, Sophia E. Schiza

**Affiliations:** 1 Pulmonology Unit, Department of Public Health and Infectious Diseases, Policlinico Umberto I, “Sapienza” University of Rome, 00185 Rome, Italy; matteo.bonini@uniroma1.it (M.B.); paolo.palange@uniroma1.it (P.P.); 2 Sleep Disorders Center, Department of Respiratory Medicine, School of Medicine, University of Crete, 70013 Heraklion, Greece; izolthi@gmail.com (I.B.); vmoniaki@yahoo.gr (V.M.); elenima23@hotmail.com (E.M.); schizas@uoc.gr (S.E.S.)

**Keywords:** obstructive sleep apnea, polysomnography, bronchial asthma, alternative overlap syndrome, fatigue, anxiety, psychology

## Abstract

Background: The alternative Overlap Syndrome (aOVS), the coexistence of bronchial asthma and Obstructive Sleep Apnea (OSA), represents a distinct clinical phenotype associated with worse clinical outcomes, but little is yet known about its characteristics. We aimed to investigate differences in sleep stability and clinical burden between OSA and aOVS patients matched for age, gender, BMI and Apnea-Hypopnea Index (AHI). Methods: 45 aOVS and 45 OSA patients were compared using demographic, clinical and polysomnographic data. Results: Patients with aOVS exhibited significantly higher odds ratio product (ORP) values for total sleep time (ORPmed: 0.8 ± 0.2 vs. 0.5 ± 0.1, *p* < 0.001) and Non-Rapid Eye Movement (ORPnr: 0.7 ± 0.3 vs. 0.4 ± 0.1, *p* < 0.001) sleep compared with OSA patients. Furthermore, patients with aOVS showed a significantly higher risk of developing clinically significant anxiety and fatigue, showing a significantly higher General Anxiety Disorder-7 (GAD-7: 8.7 ± 5.6 vs. 5.7 ± 4.7, *p* = 0.02) and significantly higher prevalence of fatigue (71% vs. 41%, *p* = 0.01). These associations remained significant after multivariable adjustment and were independent of OSA severity (AHI). Conclusions: Our findings support the concept that aOVS is characterised by significantly more unstable sleep and a greater psychological burden, even after matching with OSA patients for age, gender, BMI and AHI. Our study also highlights the need to integrate traditional sleep measures with more recent ones, such as ORP, in order to better capture the multidimensional burden of aOVS.

## 1. Introduction

Obstructive sleep apnea (OSA) is a chronic disease characterized by episodes of partial or total collapse of the upper airway during sleep. These episodes result in chronic intermittent hypoxia (CIH), sleep fragmentation with nocturnal awakenings, changes in intrathoracic pressure, and nocturnal fluctuations in the autonomic nervous system [1,2,3,4]. All of these OSA-related issues are linked to the now well-known increased risk of cardiometabolic events, neurocognitive impairment, car accidents caused by excessive daytime sleepiness in patients presented with this phenotype, and high healthcare costs [5,6,7,8]. In addition, OSA is a chronic condition with a high prevalence; recent estimates indicate a global prevalence of between 17% and 30% among people aged 30–69, rising to as high as 50% among those over 70 years [9,10]. Another key point of scientific debate is the clinical heterogeneity of OSA. Patients with the same OSA severity, as determined by Apnea-Hypopnea Index (AHI), may exhibit completely different symptoms, comorbidities, and long-term cardiometabolic risks [11]. Regarding this heterogeneity, there has been much discussion in recent years about OSA phenotypes, identified through cluster analysis of comorbidities and clinical and polysomnographic features. The most identified phenotypes are: the excessively sleepy phenotype with increased cardiometabolic risk, the minimally symptomatic phenotype that remains undiagnosed for several years, and finally, the disturbed sleep with features of insomnia phenotype [12]. Being able to identify these phenotypes—whose characteristics go beyond the severity of AHI—enables the management of these patients by stratifying them according to the impact of the condition on their quality of life, their risk of future cardiovascular events, and their clinical presentation. However, the phenotypes currently used in the literature have limitations. They are based on cluster analyses that may not account for geographical distribution, ethnicity, gender, socioeconomic status, and environmental factors. Consequently, it is important to emphasize the need to improve the current phenotyping of OSA so that it can truly capture its real-world heterogeneity and multidimensional burden. For this reason, several new metrics have been developed in recent years to better capture the multidimensional burden of OSA. One of the most important is certainly the hypoxic burden (HB), which measures the depth, duration, and amplitude of event-related desaturations and has been shown to correlate more strongly than the AHI with cardiovascular events in individuals with OSA [13]. Autonomic activation indices, such as Pulse Wave Amplitude Drop (PWAD), have also been shown to better explain cardiovascular risk in OSA patients, given their role in reflecting nocturnal sympathetic hyperactivity [14]. New electroencephalographic metrics have been developed, such as the Arousal Burden, which represents the total duration of sleep spent in a state of arousal; when elevated, it is associated with an increased risk of cardiovascular events and long-term mortality [15]. Another recent electroencephalographic metric for sleep stability and depth detection is the Odds Ratio Product (ORP), which has been shown to be a better predictor of Comorbid Insomnia and Sleep Apnea (COMISA) than traditional sleep metrics [16]. However, these metrics have not yet been universally validated, and the measurement methodology is not standardised either. Comorbidities also contribute to the variability of the multidimensional OSA burden, and chronic respiratory conditions—including asthma—are certainly among the most significant. People with bronchial asthma are at greater risk of developing OSA, and vice versa. This bidirectional relationship is due in part to shared risk factors (chronic rhinosinusitis, gastroesophageal reflux, and obesity) and in part to mechanical factors such as nocturnal intrathoracic pressure changes and systemic inflammatory responses [17]. The coexistence of Asthma and OSA, so-called Alternative Overlap Syndrome (aOVS), is still frequently underdiagnosed. aOVS represents a distinct endophenotype, with nocturnal bronchial hyperresponsiveness, altered ventilatory control, autonomic nervous system dysregulation, and a lower arousal threshold [17]. From a clinical perspective, patients with aOVS are more likely to have less well-controlled asthma, with more frequent exacerbations and a lower quality of life; however, these issues improve with the use of CPAP [17,18,19,20]. These characteristics of OSA during sleep affect the sleep quality of these patients, leading to sleep fragmentation. Previous studies have shown a higher prevalence of the “low arousal threshold” endotype in aOVS, resulting in microarousals and fragmented sleep [21]. These pathophysiological data lead us to hypothesize that patients with aOVS, regardless of the severity of their OSA, experience a clinical burden, particularly a psychological one. For this reason, we set out to test our hypothesis: do patients with aOVS, regardless of the severity of their OSA, BMI, gender, and age, experience a greater psychological burden more frequently? However, whether aOVS is associated with specific alterations in sleep stability and psychological burden independently of respiratory event severity remains largely unexplored.

In this context, the primary objective of this study was to investigate differences in sleep stability and psychological burden between patients with aOVS and patients with OSA alone, matched for age, gender, BMI, and AHI.

Specifically, we aimed to: (1) compare objective markers of sleep instability using the Odds Ratio Product (ORP), including ORPmed and ORPnr; (2) assess psychological burden using validated questionnaires, with a focus on anxiety (GAD-7) and fatigue (FSS); (3) compare polysomnographic and clinical parameters between the two groups; and (4) evaluate the independent association of aOVS with sleep instability and psychological burden using multivariable regression models.

We hypothesized that patients with aOVS would exhibit greater sleep instability and increased psychological burden compared to patients with OSA alone, independently of OSA severity and other potential confounders.

## 2. Materials and Methods

### 2.1. Study Design

We conducted a cross-sectional, matched case–control study at the Sleep Disorders Center in the Respiratory Medicine Department of the University of Crete between July 2024 and July 2025. Participants were consecutively recruited among patients referred to the center during the study period, representing a non-probabilistic sampling strategy. The study included 90 adult subjects diagnosed with OSA, including 45 patients with aOVS and 45 patients with OSA-only. Patients were matched 1:1 between OSA and aOVS, according to age, gender, BMI and AHI. Sample size was estimated using ORPmed as the primary endpoint, assuming a relevant difference of 0.2 between aOVS and OSA, based on prior literature indicating that relatively small variations in ORP reflect meaningful differences in sleep stability across physiological sleep states and clinical phenotypes [16]. Ethical approval (approval number 7370/date 4 June 2014) was granted by the Hospital Ethics Committee of the University of Crete, and all participants provided written informed consent. The inclusion criteria included: age ≥ 18 years old, OSA confirmed by polysomnography (PSG) with AHI ≥ 5 events/h and for only the aOVS a positive remote pathological history for BA, with diagnosis made in accordance with recent international guidelines based on the patient’s clinical history or the recurrence of signs and symptoms typical of BA, in combination with evidence of variable expiratory flow in pulmonary function tests (PFT) [22]. Exclusion criteria included a diagnosis of other chronic respiratory diseases, neuromuscular disorders, Obesity-Hypoventilation Syndrome (OHS), non-respiratory sleep disorders, psychiatric disorders, severe cognitive impairment, drug/alcohol abuse and Asthma Control Test (ACT) score < 20 at the first visit to our centre or an asthma exacerbation in the 4 weeks prior to PSG (this last one only for aOVS group). All patients were treated with inhaled corticosteroid/long-acting β2-agonist (ICS/LABA) therapy, and none were receiving biologic treatments.

### 2.2. Data Collected

We collected data from the first medical visit such as age, gender, BMI, smoking and alcohol habits, comorbidities, symptoms, spirometry (FEV_1_%, FVC% and FEV1/FVC). We also collected the following questionnaires concerning daytime sleepiness, features of insomnia, psychological burden and fatigue:

1. Epworth Sleepiness Scale (ESS) to define excessive daytime sleepiness (EDS) with ESS > 10 points [23]
2. Athens Insomnia Scale (AIS) to define insomnia (≥6 points) [24]
3. Patient Health Questionnaire-9 (PHQ-9) to define depression (>9 points) [25]
4. General Anxiety Disorder-7 (GAD-7) to define anxiety (>4 points) [26]
5. Pittsburgh Sleep Quality Index (PSQI) to define poor sleep quality (>5 points) [27]
6. Fatigue Severity Scale (FSS) to define fatigue (>36 points) [28]

We also collected data about subjective markers of sleep instability: subjective sleep latency, referred frequent nocturnal awakenings and use of hypnotic medications.

### 2.3. Polysomnography

All patients underwent overnight in-lab PSG for suspected OSA. PSG was performed with Alice 5 Diagnostics (Respironics, Murrysville, PA, USA, software 2.8.78), according to the standard protocols of the American Academy of Sleep Medicine (AASM) [29]. The PSG included the following integrated channels: three-lead EEG (frontal, central, and occipital), electrooculogram, electromyogram, flow (using an oronasal thermistor and nasal pressure transducer), chest and abdominal effort, oxygen saturation (SpO_2_), heart rate, PPG, electrocardiogram (ECG) using lead II configuration, snoring (using a microphone on the front of the neck), and body activity. Apnoea and hypopnoea were assessed according to AASM guidelines. Apnoea was defined as when the oronasal thermistor flow was reduced by at least 90% from the pre-event baseline for at least 10 s [29]. Hypopnoea was defined as a reduction in nasal airflow of at least 30% from the pre-event baseline for at least 10 s, in combination with a desaturation of ≥3% or an EEG arousal [30].

The PSG analysis included:

1. Objective markers of sleep instability: We used the odds ratio product (ORP), a validated continuous index derived from EEG spectral analysis that measures every 3 s the sleep depth using a continuous scale from 0 (very deep sleep) to 2.5 (full wakefulness) from the central lead EEG [30]. We analyzed ORPmed (the median ORP during the entire recording) and ORPnr (the median ORP during NREM sleep). Higher ORP values indicate sleep instability, whereas lower ORP values indicate deeper sleep [31].
2. Sleep quality parameters: total sleep time (TST), sleep efficiency (defined as the ratio of TST to sleep period), wake after sleep onset (WASO) or total wake time between sleep onset and final awakening, the percentage of slow-wave sleep (SWS), non-rapid eye movement (NREM) and rapid-eye movement (REM) sleep on TST, N2 phase latency, REM phase latency, arousal index (AI), REM-AI, NREM-AI.
3. Respiratory parameters: AHI, REM-AHI, NREM-AHI, average apnoea duration, average hypopnoea duration, oxygen desaturation index (ODI), average SpO_2_, minimum SpO_2_, sleep time spent with SpO_2_ < 90% (T90%).

### 2.4. Statistical Analysis

Sample size was estimated using ORPmed as the primary endpoint, assuming a relevant difference of 0.2 between aOVS and OSA, based on prior literature indicating that relatively small variations in ORP reflect meaningful differences in sleep stability across physiological sleep states and clinical phenotypes [16]. Using a two-sided alpha level of 0.05 and a 90% of statistical power, at least 30 patients per group were required. Therefore, the final number of our cohort (45 for each group) was considered sufficient for the statistical analysis. Continuous variables were reported as mean ± standard deviation. Categorical variables were summarized as counts and percentages. Baseline characteristics were compared between groups using Student’s *t*-test, Mann–Whitney U test, or χ^2^/Fisher’s exact test, depending on data distribution. We used multivariable regression models to evaluate the impact of aOVS on sleep instability and clinical burden, reporting β coefficients with 95% confidence intervals for linear models and odds ratios (OR) with 95% confidence intervals for logistic models. Although matching was performed on age, gender, BMI, and AHI, these variables were retained in multivariable models to account for potential residual imbalance after matching and to improve the estimate precision. A two-sided *p*-value < 0.05 was considered statistically significant.

## 3. Results

### 3.1. Baseline Characteristics

The study included 90 patients, comprising 45 with OSA and 45 with aOVS (Table 1). The gender distribution varied little within the cohort (55% men and 45% women for both groups). The prevalence of major cardiometabolic comorbidities did not differ statistically significantly between the two groups, except for cerebrovascular diseases, which were significantly more frequent in the OSA group compared to the aOVS group (11% vs. 0%, *p* = 0.04). Coronary heart disease (CHD) was also more prevalent in the OSA group (13%) with respect to the aOVS group (2%), although this difference did not reach statistical significance (*p* = 0.1). Regarding symptoms, nocturnal snoring and non-restorative sleep were highly prevalent in both groups (96% and 87% for OSA, 96% and 93% for aOVS). Morning headaches were frequently more reported in OSA than in aOVS patients (69% vs. 42%, *p* = 0.02), whereas the prevalence of frequent nocturnal awakenings was higher in aOVS (75% vs. 55%), although this difference did not reach statistical significance (*p* = 0.07). No significant differences were observed in nocturia (71% vs. 75%). The ESS did not differ significantly between the two groups, while the use of hypnotics before bedtime was more common among aOVS patients than among OSA patients, though it did not reach statistical significance (18% vs. 5%, *p* = 0.08).

### 3.2. Psychological Burden

Anxiety severity measured by the GAD-7 scale was significantly higher in patients with aOVS compared with OSA (8.7 ± 5.6 vs. 5.7 ± 4.7, *p* = 0.02). Consequently, the prevalence of anxiety disorder was significantly higher in the aOVS group (77% vs. 51%, *p* = 0.04). Fatigue severity measured by FSS was higher in aOVS patients without reaching statistical significance (42 ± 14 vs. 36.3 ± 15.8, *p* = 0.1), whereas the prevalence of clinically relevant fatigue was significantly greater in the aOVS group (71% vs. 41%, *p* = 0.01). Features of depression and insomnia, measured, respectively, by PHQ-9 and AIS, were comparable between OSA and aOVS. Sleep quality (PSQI) did not differ significantly between the two groups.

### 3.3. Sleep Instability

Polysomnographic features were largely comparable between OSA and aOVS (Table 2). Indices of sleep efficiency, sleep fragmentation, and apneic severity were similar between the two groups. However, ORP, an electroencephalographic metric of sleep stability, differed significantly between the two groups. Both ORPmed and ORPnr were significantly higher in aOVS patients compared with OSA controls (0.8 ± 0.2 vs. 0.5 ± 0.1 and 0.7 ± 0.3 vs. 0.4 ± 0.1, respectively; both *p* < 0.001).

### 3.4. Multivariate Analysis

We used multivariate linear regression analysis to determine whether aOVS was associated with a greater sleep instability and psychological burden even after adjusting for age, BMI, AHI, and T90% (Table 3). The adjusted models showed that aOVS remained significantly associated with 0.329-unit higher ORPmed (95% CI 0.237–0.421, *p* < 0.001) and a 0.317-unit higher ORPnr (95% CI 0.213–0.421, *p* < 0.001) compared to OSA. Regarding psychological burden, aOVS patients had significantly higher anxiety scores by GAD-7 (β = 3.472, 95% CI 0.681–6.264, *p* = 0.015) and higher fatigue severity by FSS (β = 7.916, 95% CI 0.163–15.670, *p* = 0.045).

The multivariable logistic regression analysis further demonstrated an increased risk of abnormal psychological distress in aOVS patients (Table 4). The adjusted models showed that aOVS was independently associated with higher odds of clinically relevant anxiety (GAD-7 > 4; OR 4.43, 95% CI 1.35–14.55, *p* = 0.01) and fatigue (FSS > 36; OR 4.45, 95% CI 1.42–13.98, *p* = 0.01) compared with OSA. By contrast, aOVS was not significantly associated with insomnia, poor sleep quality or EDS.

## 4. Discussion

In this study, we investigated differences in sleep stability and psychological burden between patients with OVS and OSA, matched for gender, age, BMI, and AHI. We can summarize our main findings in three key points. The first observation is that patients with aOVS experienced a greater psychological burden, especially regarding anxiety and fatigue. The second observation is that aOVS patients exhibited significantly greater sleep instability, despite similar demographic characteristics and similar severity of respiratory events. The third observation is that these differences observed in patients with aOVS may therefore suggest the existence of a distinct clinical phenotype for aOVS characterized by a multidimensional burden of the condition that cannot be fully captured by traditional sleep indices alone.

### 4.1. The Psychological Burden

The existence of a distinct clinical phenotype for aOVS characterized by a greater psychological burden is consistent with previous studies suggesting that these patients may have poorly controlled asthma, with more exacerbations and a reduced quality of life [17,18,19,20]. More specifically, aOVS patients exhibit low scores on most Patient-Reported Outcomes Measures (PROMs), including Asthma Control Test (ACT), Leicester Cough Questionnaire (LCQ), COPD Assessment Test (CAT), and Asthma Health Questionnaire-33 (AHQ-33) [32]. Importantly, the association between aOVS and psychological burden remained significant after multivariable adjustment, suggesting that the increased psychological burden observed in aOVS is not solely explained by differences in OSA severity or nocturnal hypoxemia. Previous studies have shown that sleep quality is impaired in a significant proportion of asthmatic patients, especially when asthma is poorly controlled or when it is associated with comorbidities such as OSA or Gastro-Esophageal Reflux Disease (GERD). Data in the literature regarding the psychological impact of OSA in patients with asthma are scarce, but some evidence does exist. Davies SE et al. demonstrated an improvement in quality-of-life scores among patients with obstructive sleep apnea (OSA) treated with Continuous Positive Airway Pressure (CPAP) [33]. They also demonstrated an improvement in daytime fatigue and in the vitality domain of the 36-item short form (SF-36) questionnaire after 3 months of CPAP follow-up in patients with OSA [34]. Previous studies have also demonstrated that uncontrolled asthma has been associated with a 3.3-fold increased risk of poor sleep quality (Pittsburgh Sleep Quality Index score > 5), a 1.72-fold increased risk of initial insomnia, and a 1.55-fold increased risk of sleep maintenance insomnia [35,36].

### 4.2. The Sleep Instability

Our finding regarding greater sleep instability in patients with aOVS, as evidenced by higher levels of ORPmed and ORPnr, may be explained by the pathophysiological mechanism through which asthma can contribute to nocturnal bronchial hyperresponsiveness and increased airway resistance, which may contribute to frequent awakenings and fragmented sleep [17]. The low arousal threshold, which has been reported to be more prevalent in aOVS patients, may predispose to micro-arousals and fragmented sleep [21]. It is also known that low arousal threshold is associated with a higher prevalence of neurosymptomatic manifestations such as depression, anxiety, fatigue, and insomnia [37]. Regarding electroencephalographic findings, a previous study showed that patients with aOVS spent more time in N1 and N2 sleep and less time in REM sleep and deep sleep (N3) [38]. Notably, the higher ORP values observed in aOVS patients remained independently associated with the overlap condition after adjustment for major confounders, reinforcing the concept of a distinct pathophysiological phenotype. Furthermore, previous studies have shown that patients with aOVS exhibit lower mean nocturnal oxygen saturation and that asthma control deteriorated as T90% increased, suggesting a possible correlation between nocturnal hypoxemia and asthma control [38,39]. Small airway disease (SAD) has also been reported as an independent predictor of T90% in patients with OSA [40]. However, there are no other studies in the literature that compare nocturnal electroencephalographic features, nor ORP, between OSA and aOVS.

### 4.3. Clinical Implications

From a clinical perspective, our findings suggest that AHI alone may not fully capture the burden of aOVS. In fact, our study showed that, despite having similar AHI, BMI, age, and gender, patients with aOVS exhibited more unstable sleep and a greater psychological burden, characterized by higher levels of anxiety and fatigue. Although no direct data are available in the literature regarding the psychological burden and the predictive role of AHI in aOVS, several studies conducted on patients with OSA have demonstrated a weak correlation between AHI severity and anxiety or depression. Some cohort studies have shown that patients with severe OSA (high AHI) scored lower on anxiety and depression scales than patients with mild OSA [41,42]. Fatigue is not well predicted by the AHI in OSA patients, whereas it is more frequently associated with EDS and with the COMISA phenotype. Insomnia itself is more common in mild OSA, according to some studies [42,43]. This limitation of AHI in capturing the psychological burden of OSA may reflect underlying pathophysiological factors, such as sleep fragmentation, intermittent hypoxia, and systemic inflammation characterized by elevated cytokine levels and high oxidative stress [44,45]. Consequently, our results suggest that relying solely on the AHI to assess the psychological burden of patients with OSA and aOVS may be insufficient; instead, the evaluation of these patients should be integrated into a multidisciplinary approach that includes both psychological data and objective measures of sleep quality and stability.

### 4.4. Study Strengths and Limitations

The study has several strengths. The first is that patients with OVS and those with OSA alone were matched for age, gender, BMI, and AHI, thereby minimizing confounding factors when analyzing differences between these two populations. The second is the multidimensional assessment of the psychological burden of these patients, using recent metrics such as the ORP as well as subjective scales for anxiety, depression, insomnia, and fatigue. However, the study also has some limitations. First, the cross-sectional design precludes causal inference between aOVS, sleep instability, and psychological burden. For this reason, future longitudinal studies are needed. Second, the study is single-center, which may limit the generalizability of our findings. Third, our study population consisted of patients with well-controlled asthma (ACT ≥ 20), which may limit generalizability to real-world aOVS populations, where asthma may be poorly controlled. Fourth, we did not include detailed data on asthma phenotyping or inflammatory profiles, which may influence both sleep quality and psychological burden. However, all patients were receiving ICS/LABA therapy, and none were treated with biologics, partially reducing treatment-related variability. Finally, although the minimum required sample size was exceeded, the number of enrolled patients is still modest, so the results should be interpreted with caution.

## 5. Conclusions

aOVS remains an understudied and underdiagnosed condition, despite its clinical complexity and high prevalence of the two conditions of both asthma and OSA. In this study, patients with aOVS exhibited greater sleep instability, as reflected by higher ORP values, and a greater psychological burden, particularly in terms of anxiety and fatigue, despite similar OSA severity and considering that asthma was not poorly controlled (ACT > 20 as an inclusion criterion). Furthermore, these differences remained independent of major confounders. These findings support the concept of aOVS as a specific clinical phenotype in which it is essential to look beyond the AHI and traditional sleep indices to identify the multidimensional burden of aOVS, paying particular attention to psychophysical well-being. Future longitudinal studies involving large cohorts are needed, but it is clear that a multidisciplinary approach is required to capture the true multidimensional burden of aOVS, with the support of new electroencephalographic metrics such as ORP.

## Figures and Tables

**Table 1:** Demographic, clinical and functional characteristics.

Variable	OSA (n = 45)	aOVS (n = 45)	*p* Value
Age, years	54 ± 12	54 ± 12	1.0
Men/Women, n (%)	25 (55)/20 (45)	25 (55)/20 (45)	1.0
BMI, kg/m^2^	33 ± 7	33 ± 7	1.0
AF, n (%)	3 (7)	2 (4)	1.0
Hypertension, n (%)	18 (40)	20 (44)	0.8
T2D	5 (11)	5 (11)	1.0
Heart Failure, n (%)	1 (2)	1 (2)	1.0
CHD, n (%)	6 (13)	1 (2)	0.1
CeVD, n (%)	5 (11)	0 (0)	0.04
Hyperlipidemia, n (%)	19 (42)	17 (38)	0.8
Snoring, n (%)	43 (96)	43 (96)	1.0
Unrefreshed sleep, n (%)	39 (87)	42 (93)	0.5
Morning headaches, n (%)	31 (69)	19 (42)	0.02
Frequent awakenings, n (%)	25 (55)	34 (75)	0.07
Nocturia, n (%)	32 (71)	34 (75)	0.8
ESS	7 ± 4.7	7.5 ± 4.7	0.6
EDS, n (%)	14 (31)	14 (31)	1.0
Subjective sleep latency, min	20.5 ± 28.5	27.5 ± 36.5	0.3
Self-reported sleep duration, hours	6.2 ± 1.3	6 ± 1.3	0.7
Use of hypnotics, n (%)	2 (5)	7 (18)	0.08
Insomnia Athens Scale	7.8 ± 5.5	9.7 ± 5	0.1
Insomnia, n (%)	20/38 (53)	20/30 (67)	0.3
PHQ-9	7.7 ± 5.8	9.8 ± 6.2	0.1
Depression, n (%)	16/38 (42)	14/30 (47)	0.8
GAD-7	5.7 ± 4.7	8.7 ± 5.6	0.02
Anxiety, n (%)	19/38 (50)	23/30 (77)	0.04
PSQI	7.4 ± 4	8.7 ± 4	0.2
Bad sleep quality, n (%)	24/38 (63)	22/30 (73)	0.6
FSS	36.3 ± 15.8	42 ± 14	0.1
Fatigue, n (%)	16/38 (42)	22/30 (73)	0.01
FEV_1_%, % of predicted	90.4 ± 14	90.6 ± 14	0.9
FVC%, % of predicted	92 ± 15.6	93.5 ± 15.5	0.7
FEV_1_/FVC	80 ± 6.5	79.7 ± 9.6	0.8

Values are reported as mean ± standard deviation for continuous variables and as number (percentage) for categorical variables. Percentages were calculated on available data for variables with missing values. Comparisons between groups were performed using Welch’s *t*-test for continuous variables and Fisher’s exact test for categorical variables. A *p*-value < 0.05 was considered statistically significant. OSA, obstructive sleep apnea; aOVS, alternative Overlap Syndrome; BMI, body mass index; AF, atrial fibrillation; T2D, type 2 diabetes; CHD, coronary heart disease; CeVD, cerebrovascular disease; ESS, Epworth Sleepiness Scale; EDS, excessive daytime sleepiness; PHQ-9 = Patient Health Questionnaire-9; GAD-7 = Generalized Anxiety Disorder-7; PSQI = Pittsburgh Sleep Quality Index; FSS = Fatigue Severity Scale; FEV1, forced expiratory volume in 1 s; FVC, forced vital capacity.

**Table 2:** Polysomnographic characteristics.

Variable	OSA (n = 45)	aOVS (n = 45)	*p* Value	Effect Size (Cohen’s d)
TST, min	279.7 ± 69.8	269.6 ± 62.5	0.5	−0.15
Sleep efficiency, %	79.6 ± 16.1	78.0 ± 16.1	0.6	−0.10
WASO, min	86 ± 64.2	90.7 ± 62.9	0.7	0.07
SWS, %	7.1 ± 6.4	9.3 ± 13.8	0.3	0.20
REM, %	14.8 ± 11.3	11.4 ± 6.7	0.08	−0.37
NREM, %	85.2 ± 11.3	90.8 ± 19	0.1	0.36
Sleep onset before N2, min	46.3 ± 45.4	46.9 ± 29.6	0.9	0.02
REM latency, min	138.1 ± 78.1	146.7 ± 73.3	0.6	0.11
ORPmed	0.5 ± 0.1	0.8 ± 0.2	<0.001	1.90
ORPnr	0.4 ± 0.1	0.7 ± 0.3	<0.001	1.34
AI, events/h	23.8 ± 18.6	24.7 ± 15.7	0.8	0.05
Respiratory AI, events/h	12.3 ± 16.5	11.6 ± 10.6	0.8	−0.05
Leg Movement AI, events/h	3.7 ± 5.5	4.9 ± 6.7	0.4	0.20
Spontaneous AI, events/h	7.8 ± 7.2	8.2 ± 8.4	0.8	0.05
AHI, events/h	29.5 ± 27.1	32.6 ± 27.2	0.6	0.11
ODI, events/h	31.0 ± 32.0	33.3 ± 32.7	0.7	0.07
T90%	11.4 ± 23.7	6.0 ± 10.7	0.2	−0.29
Mean SpO_2_, %	93.3 ± 4.7	94.1 ± 2.7	0.3	0.21
Lowest SpO_2_, %	78.5 ± 14.4	82.0 ± 9.0	0.2	0.29

Values are reported as mean ± standard deviation for continuous variables and as number (percentage) for categorical variables. Comparisons between groups were performed using Welch’s *t*-test for continuous variables and Fisher’s exact test for categorical variables. A *p*-value < 0.05 was considered statistically significant. Effect size is reported as Cohen’s d and interpreted as small (0.2), medium (0.5), and large (0.8). OSA, obstructive sleep apnea; aOVS, alternative Overlap Syndrome; TST, total sleep time; WASO, wake after sleep onset; SWS, slow-wave sleep; REM, rapid eye movement sleep; NREM, non–rapid eye movement sleep; ORPmed, median odds ratio product; ORPnr, non-REM odds ratio product; AI, arousal index; AHI, apnea–hypopnea index; ODI, oxygen desaturation index; T90, percentage of total sleep time with oxygen saturation below 90%; SpO_2_, peripheral oxygen saturation.

**Table 3:** Multivariable linear regression models assessing the association between aOVS and sleep instability, psychological burden and sleep architecture.

Outcome	*β* for aOVS vs. OSA	95% CI	*p* Value
ORPmed	0.329	0.237–0.421	<0.001
ORPnr	0.317	0.213–0.421	<0.001
GAD-7	3.472	0.681–6.264	0.015
FSS	7.916	0.163–15.670	0.045
Insomnia Athens Scale	1.655	−0.909–4.220	0.2
PSQI	1.519	−0.520–3.558	0.15
ESS	0.685	−1.300–2.670	0.5
Subjective sleep latency	6.383	−6.465–19.231	0.3
TST	−9.41	−46.2–27.4	0.6
Sleep efficiency	−1.65	−8.50–5.20	0.6
WASO	3.12	−16.9–23.1	0.7
REM latency	12.48	−18.6–43.6	0.4
REM%	−1.11	−4.9–2.7	0.5
NREM%	1.18	−2.6–5.0	0.5
SWS%	−0.92	−3.9–2.0	0.5

Linear regression models were used for continuous outcomes. Primary pathology was entered as a binary variable (aOVS vs. OSA), with OSA used as the reference category. Therefore, regression coefficients (β) represent the adjusted difference between aOVS and OSA. All models were adjusted for age, apnea–hypopnea index (AHI), and percentage of sleep time with oxygen saturation below 90% (T90%). Results are presented with 95% confidence intervals (CI). A *p*-value < 0.05 was considered statistically significant. ORPmed, median odds ratio product; ORPnr, non–rapid eye movement odds ratio product; GAD-7, Generalized Anxiety Disorder-7 scale; FSS, Fatigue Severity Scale; PSQI, Pittsburgh Sleep Quality Index; ESS, Epworth Sleepiness Scale; TST, total sleep time; WASO, wake after sleep onset; REM, rapid eye movement sleep; NREM, non-rapid eye movement sleep; SWS, slow-wave sleep.

**Table 4:** Multivariable logistic regression models assessing the association between aOVS and psychological burden.

Outcome	OR for aOVS vs. OSA	95% CI	*p* Value
Anxiety (GAD-7 > 4)	4.43	1.35–14.55	0.01
Fatigue (FSS > 36)	4.45	1.42–13.98	0.01
Insomnia (AIS > 6)	2.07	0.71–6.04	0.2
Bad sleep quality (PSQI > 5)	1.69	0.54–5.28	0.4
EDS (ESS > 10)	1.24	0.46–3.33	0.7

Logistic regression models were used for binary clinical outcomes. Primary pathology was entered as a binary variable (aOVS vs. OSA), with OSA used as the reference category. Odds ratios (OR), therefore, represent the odds of the outcome in patients with aOVS relative to those with OSA. All models were adjusted for age, apnea–hypopnea index (AHI), and percentage of sleep time with oxygen saturation below 90% (T90%). Results are presented with 95% confidence intervals (CI). A *p*-value < 0.05 was considered statistically significant. GAD-7, Generalized Anxiety Disorder-7 scale; FSS, Fatigue Severity Scale; AIS, Athens Insomnia Scale; PSQI, Pittsburgh Sleep Quality Index; EDS, Excessive Daytime Sleepiness; ESS, Epworth Sleepiness Scale.

## Data Availability

The data that support the findings of this study are available from the corresponding author upon reasonable request.

## Abbreviations

AASM	American Academy of Sleep Medicine
ACT	Asthma Control Test
AF	Atrial Fibrillation
AHI	Apnea–Hypopnea Index
AI	Arousal Index
AIS	Athens Insomnia Scale
AHQ-33	Asthma Health Questionnaire-33
aOVS	Asthma–Obstructive Sleep Apnea Overlap Syndrome
BA	Bronchial Asthma
BMI	Body Mass Index
CAT	COPD Assessment Test
CeVD	Cerebrovascular Disease
CHD	Coronary Heart Disease
CI	Confidence Interval
CIH	Chronic Intermittent Hypoxia
COMISA	Comorbid Insomnia and Sleep Apnea
CPAP	Continuous Positive Airway Pressure
ECG	Electrocardiogram
EDS	Excessive Daytime Sleepiness
EEG	Electroencephalogram
ESS	Epworth Sleepiness Scale
FEV1	Forced Expiratory Volume in 1 Second
FSS	Fatigue Severity Scale
FVC	Forced Vital Capacity
GAD-7	Generalized Anxiety Disorder-7
HB	Hypoxic Burden
ICS	Inhaled Corticosteroids
LABA	Long-Acting Beta-2 Agonists
LCQ	Leicester Cough Questionnaire
NREM	Non–Rapid Eye Movement Sleep
ODI	Oxygen Desaturation Index
OHS	Obesity-Hypoventilation Syndrome
OR	Odds Ratio
ORP	Odds Ratio Product
ORPmed	Median Odds Ratio Product
ORPnr	Odds Ratio Product during NREM Sleep
OSA	Obstructive Sleep Apnea
PFT	Pulmonary Function Tests
PHQ-9	Patient Health Questionnaire-9
PPG	Photoplethysmography
PROMs	Patient-Reported Outcome Measures
PSG	Polysomnography
PSQI	Pittsburgh Sleep Quality Index
PWAD	Pulse Wave Amplitude Drop
REM	Rapid Eye Movement Sleep
SAD	Small Airway Disease
SF-36	36-Item Short Form Health Survey
SpO_2_	Peripheral Oxygen Saturation
SWS	Slow-Wave Sleep
T2D	Type 2 Diabetes
T90%	Percentage of Sleep Time with SpO_2_ < 90%
TST	Total Sleep Time
WASO	Wake After Sleep Onset
